# Surfactants for Bubble Removal against Buoyancy

**DOI:** 10.1038/srep19113

**Published:** 2016-01-08

**Authors:** Md. Qaisar Raza, Nirbhay Kumar, Rishi Raj

**Affiliations:** 1 Thermal and Fluid Transport Laboratory, Department of Mechanical Engineering, Indian Institute of Technology, Patna, Bihar 801103, India

## Abstract

The common phenomenon of buoyancy-induced vapor bubble lift-off from a heated surface is of importance to many areas of science and technology. In the absence of buoyancy in zero gravity of space, non-departing bubbles coalesce to form a big dry patch on the heated surface and heat transfer deteriorates despite the high latent heat of vaporization of water. The situation is worse on an inverted heater in earth gravity where both buoyancy and surface tension act upwards to oppose bubble removal. Here we report a robust passive technique which uses surfactants found in common soaps and detergents to avoid coalescence and remove bubbles downwards, away from an inverted heater. A force balance model is developed to demonstrate that the force of repulsion resulting from the interaction of surfactants adsorbed at the neighboring liquid-vapor interfaces of the thin liquid film contained between bubbles is strong enough to overcome buoyancy and surface tension. Bubble removal frequencies in excess of ten Hz resulted in more than twofold enhancement in heat transfer in comparison to pure water. We believe that this novel bubble removal mechanism opens up opportunities for designing boiling-based systems for space applications.

Boiling is a ubiquitous physical process governing many day-to-day activities such as cooking, water purification, and industrial applications including thermoelectric power generation, cooling of electronic equipment, chemical and petrochemical processes, cryogenic fuel storage, refrigeration and air conditioning^1^,^2^,^3^,^4^. In comparison to other modes of heat transfer such as single-phase air or liquid cooling which rely on the low sensible heat of fluid, boiling utilizes the relatively high latent heat of vaporization to enable efficient heat transfer at relatively small temperature budgets. Liquid close to the heated surface evaporates to form vapor bubbles. A vapor bubble experiences two types of opposing forces governing its departure from the surface (Fig. 1a), namely, buoyancy (red arrow) acting upwards (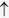), and the component of surface tension force (green arrow) acting downwards (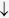).The surface tension force is proportional to *σR* where *σ* is the surface tension of the fluid, and *R* is the radius of spherical cap bubble. Buoyancy is proportional to 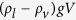 where *ρ*_*l*_ is the density of liquid, *ρ*_*v*_ is the density of vapor, *g* is the acceleration due to gravity, and 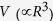 is the volume of the bubble. With increase in size above a critical radius, buoyancy (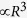) overcomes the surface tension force (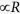) and the bubble detaches from the heated surface^5^. The departing bubbles travel away to release the associated thermal energy to a sink *via* condensation while the surface is rewet by the surrounding cold fluid. The ebullition cycle comprising of nucleation, bubble growth, removal/departure, and rewetting continues, with heat transfer increasing with bubble departure frequency^6^.

In the absence of gravity (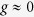), the boiling behaviour is significantly altered^7^,^8^ and the advantage of high heat transfer associated with boiling on earth is lost in space due to the weak gravitational forces^2^,^9^,^10^,^11^,^12^,^13^. The ebullition cycle is eliminated and nucleating bubbles coalesce to form a large primary bubble which is held on to the heated surface by surface tension forces. The boiling heat transfer deteriorates due to the formation of a large dry patch underneath the primary^2^,^7^,^8^,^10^,^11^,^12^,^13^,^14^ bubble (Fig. 1b), and as a result, thermal management of space-based systems (Mercury, Gemini, Apollo, MIR Space Station, US Space Shuttle, Russian Soyuz Spacecraft, and the International Space Station) are typically (except for heat pipes) addressed using conventional single-phase cooling strategies. The use of single-phase solutions results in low energy-to-mass ratios of the cooling device and high launch and maintenance costs are incurred^15^.

Increasing international activities accompanied by the corresponding increase in the size and power requirements of space-based infrastructure demand improvements in the energy-to-mass ratio *via* the incorporation of multiphase systems. Any advancement made in this direction is also expected to be instrumental in alleviating the problem of low heat transfer associated with the absence of bubble removal in confined spaces as well as on inverted (downward facing) heaters in earth gravity. While electrical, vibration, and acoustic excitations^16^,^17^ have been proposed as an alternative to induce bubble removal in the absence of buoyancy, they increase the system complexity, are energy intensive, and often compromise reliability. For example, the use of electric fields imposes the threat of dielectric breakdown of water^18^. Conversely, dielectric fluids proposed to address the problem of dielectric breakdown have very small latent heat of vaporization in comparison to water.

In this work, we present a completely passive mechanism of bubble removal against buoyancy on an inverted heater setup (−*ve* gravitational component *w.r.t.* heater orientation) in a laboratory environment (Supplementary Fig. 1 and Methods). Both surface tension and buoyancy forces act upwards (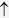) and push bubbles towards the inverted heater surface. In the absence of departure, bubbles on the heater gradually coalesce to form a large primary water vapor bubble. The resulting dry patch underneath the big vapour bubble (Fig. 1c, Supplementary Movie 1) lowers the heat transfer, very similar to what happens in zero gravity^8^,^12^ (Fig. 1b). The key innovation here lies in the use of aqueous surfactant solutions to avoid bubble coalescence^19^,^20^,^21^ (Fig. 1d and the image in the inset). Since surfactants also increase the nucleation site density^22^,^23^, multiple smaller non-coalescing bubbles were formed and the dry patches were significantly reduced upon using the various types of aqueous surfactant solutions as the test fluid (Fig. 1d). Moreover, these fast growing nucleating bubbles in wet patches were observed to force the surrounding large bubbles away from the heated surface (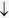), even against the combined effect of buoyancy and surface tension force (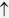) (Supplementary Movie 2). A maximum bubble removal/departure frequency of ≈15–16 Hz was observed with surfactant solutions. Maximum heat flux *q*″ (heat transfer per unit area) of ≈500 kW/m^2^ and heat transfer coefficient (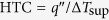, heater transfer per unit area per degree temperature rise above saturation) of ≈36 kW/m^2^-K is reported. These values correspond to ≈140% enhancement in both heat flux and heat transfer coefficient in comparison to pure water on an inverted heater.

## Bubble Departure

**Figure 1:**
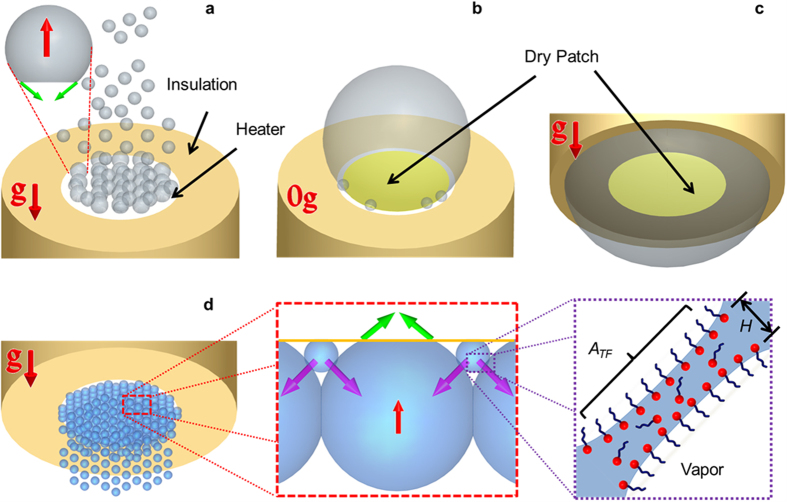
Comparison of bubble behavior at earth and in zero gravity. (**a**) Boiling of water on an upward facing heater in earth gravity. Nucleating bubbles all around the heater grow in size, buoyancy (red arrow) overcomes surface tension force (green arrow), and bubble removal is observed. (**b**) Boiling of water in zero gravity. Buoyancy is absent, bubble departure is not observed, and a large dry patch is formed on the heated surface. (**c**) Boiling of water on an inverted heater in earth gravity (−1 *g*). Both surface tension and buoyancy forces act together to retain bubbles on the heater surface. Non-departing bubbles coalesce to form a large bubble with significant dry patch similar to (**b**). (**d**) Boiling with aqueous surfactant solution on an inverted heater in earth gravity. Coalescence is minimized (image in inset) and multiple smaller bubbles are formed. Surface tension force (green arrow) and buoyancy (red arrow) on the relatively big bubble is overcome by the force of repulsion (purple arrow) provided by the fast growing satellite bubbles to facilitate departure. The force of repulsion originates from the interaction (image in the inset) between the surfactants (red dot is hydrophilic head and blue chain is hydrophobic tail) adsorbed at the neighboring liquid-vapor interfaces of the thin liquid film trapped between the bubbles.

**Figure 2:**
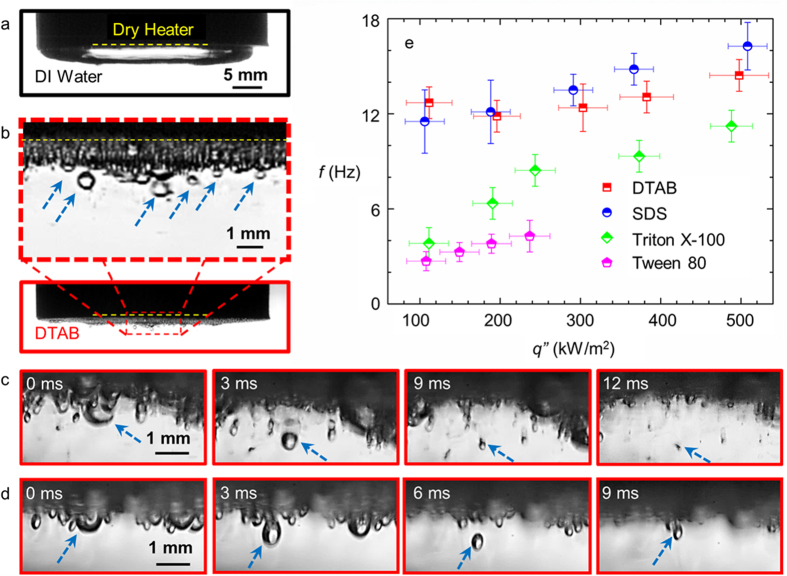
Side view images and bubble departure frequency. Side view images of boiling with (**a**) DI water (stagnant bubble) and (**b**) aqueous DTAB solution at critical micelle concentration (CMC) and a heat flux of ≈200 kW/m^2^. Surfactant induced bubble removal can be clearly seen in the image in inset (blue dotted arrows). (**c**) Time-lapse images illustrating collapse of departed bubbles due to condensation. (**d**) Departing bubble often returns to the heater surface due to buoyancy. (**e**) The corresponding plot of bubble departure frequency versus heat flux for all surfactants. Bubble removal frequency *f* was observed to increase with heat flux. The temperature of the liquid pool was maintained at 50 ± 1 °C. Note that no bubble removal was observed with water.

To study the bubble departure mechanism, boiling heat transfer experiments were conducted on a custom built experimental setup with aqueous solutions of DTAB (Dodecyltrimethylammonium bromide, cationic), SDS (Sodium Dodecyl Sulfate, anionic), Triton X-100 (Polyoxyethylated t-octylphenol, nonionic), and Tween80 (Polyethylene Sorbitan Monooleate, nonionic) as the test fluid (see Methods). High speed visualization (Vision Research, Phantom v7.3) of the boiling process from side was performed to elucidate the new bubble departure mechanism. While a large dormant coalesced bubble which covered the entire heated area was formed with water (action of buoyancy in the same direction as the surface tension force changed the bubble shape from the usual spherical cap to a compressed ellipsoidal type geometry, Figs 1c and 2a), coalescence was minimized with aqueous surfactant solutions and multiple bubbles were formed on the heater surface (image for DTAB in Fig. 2b). When a bubble nucleates, surfactant monomers from the bulk liquid gradually diffuse towards the liquid-vapor interface. The hydrophilic head of the adsorbed surfactant on the liquid-vapor interface lies in the solution while the hydrophobic tail resides in the vapor phase (Fig. 1d). When two bubbles approach each other, the head molecules at the respective interfaces repel each other and delay coalescence^19^,^20^,^21^. Electrostatic interactions dominates in ionic surfactants (DTAB and SDS) while hydrophobic interactions are important in non-ionic surfactants (Triton X-100 and Tween 80), both of which are tested here (see Methods for solution preparation details).

More importantly, surfactants facilitate a cyclic process resembling ebullition wherein the non-coalescing bubbles growing at a very rapid rate during the early stages of their life, push the surrounding bubbles away from the heated surface (Fig. 1d), resulting in bubble removal (dotted arrow, Fig. 2b) followed by rewetting. Departed bubbles come in contact with the surrounding cold fluid, shrink in size due to condensation, eventually resulting in complete collapse (Fig. 2c). It should be noted here that a few large departed bubbles while condensing in the liquid pool were often returned back to the heated surface due to the counteracting effect of buoyancy in our experiments (Fig. 2d). Accordingly, a vapor bubble stack was formed underneath the heater surface (Fig. 2b–d). However, the final size of these bubbles was much smaller than the size at departure due to condensation during the exposure to bulk liquid. Accordingly, there will be some reduction is heat transfer due to the increase in thermal resistance resulting from such vapor bubble stacks.

To quantify the departure characteristics of the bubbles, we first studied the departure frequency as a function of boiling heat flux for the different surfactants (Fig. 2e). The bubble departure frequency was observed to increase with heat flux. DTAB and SDS resulted in very high bubble departure frequency of up to ≈15–16 Hz which are comparable to the nominal departure frequency due to buoyancy in earth gravity^24^,^25^. The maximum departure frequency for Triton X-100 was ≈12 Hz while it was only ≈3–4 Hz for Tween 80 solution.

**Figure 3:**
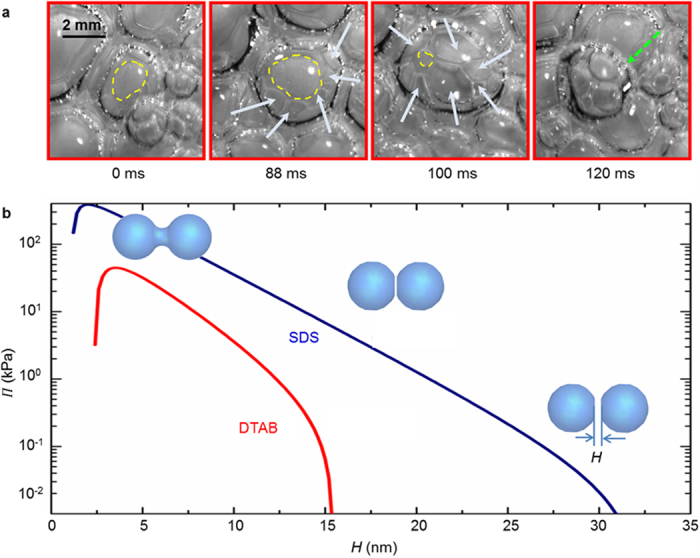
Bubble departure mechanism. (**a**) Time-lapse images from nucleation to departure during boiling on an inverted heater with aqueous DTAB solution at CMC and a heat flux of ≈200 kW/m^2^. Consider a bubble at 0 ms wherein the contact line is shown by yellow dashed curve. Multiple satellite bubbles nucleate (88 ms, white arrows) within the wedge shaped microlayer region of this bubble. These fast growing satellite bubbles apply a force away from the heater surface on the big bubble, contact line is observed to advance (100 ms, dry area shrinks), and bubble departs away from the inverted heater surface. The departed bubble is observed to shrink in size upon exposure to cooler liquid pool (green dotted arrow, 120 ms) which was maintained at 90 ± 1 °C. (**b**) Plot of the disjoining pressure with liquid film thickness for DTAB and SDS at CMC. The plateau in the disjoining pressure curve pertains to thin-film rupture resulting in bubble coalescence.

The fundamental mechanism of bubble departure during boiling with aqueous DTAB solution on a downward facing heater is illustrated through Supplementary Movie 3 (liquid pool was maintained at 50 ± 1 °C) and Supplementary Movie 4 (liquid pool was maintained at 90 ± 1 °C). Small satellite bubbles formed after the new nucleation events within the wedge shaped microlayer region of the relatively large primary bubble act as a precursor to departure (Fig. 3a). The departure mechanism is explained by considering a simple force balance model wherein surface tension and buoyancy act to retain (up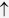) the primary bubble on the heater surface: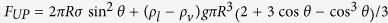, where *R* is the radius of the bubble, *σ* is the equilibrium surface tension of the aqueous surfactant solution, *g* is the acceleration due to gravity, *θ* is the contact angle, and *ρ*_*l*_ and *ρ*_*v*_ are the densities of the liquid and vapor phases. Conversely, increasing interaction of the surfactant layers due to the continuously decreasing thickness *H* of the liquid film trapped between the primary bubble and fast growing satellite bubbles (Fig. 1d, image in the inset) provide the opposing repulsive force for the departure of the primary bubble (capillary force, inertia force due to bubble growth, and drag are small and have been neglected). According to the extended Derjaguin-Landau-Verwey-Overbeek (DLVO) theory, the disjoining pressure 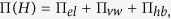 where, 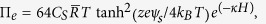 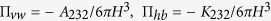
are the contribution due to electrostatic, dispersion/van der Waals, and hydrophobic interactions, respectively. Here, *C*_*S*_ is the molar concentration of the surfactant in the bulk, 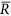 (8.314 J/mol-K) is the universal gas constant, *T* is the liquid film temperature in K, *z* is the valency of the surfactant molecule, *e* is the unit electronic charge, 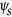 is the Stern potential at the liquid-vapor interface, 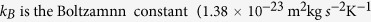), 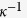
is the Debye length and depends on *C*_*S*_, *A*_232_ is the Hamaker constant for the film, and *K*_232_ is the hydrophobic force constant. The component of net repulsive force (down 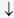) between the bubbles can then be written as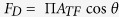, where *A*_*TF*_ is the area of the liquid-vapor interface in the thin-film region between the primary bubble and the satellite bubbles (Fig. 1d). If this component of the net repulsive force exceeds the combination of surface tension and buoyancy (*F*_*D*_ > *F*_*UP*_) before the liquid film thickness is reduced to the critical film thickness *H*_*CR*_ for coalescence/rupture of the thin-film (plateau in the disjoining pressure curve), bubble departure is facilitated. It should be noted here that the repulsive interactions discussed here are similar to those deemed responsible for the stability of foams (against coalescence) in literature^26^,^27^,^28^,^29^.

An example calculation of the disjoining pressure in the thin-film region versus the liquid film thickness for aqueous DTAB and SDS solution at CMC is illustrated in Fig. 3b. The values of various parameters required to estimate the disjoining pressure^26^,^27^ are reported in Supplementary Table S2. Considering the sample case of the bubble in Supplementary Movie 4 where the bubble departure radius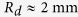, contact angle 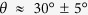 (estimated from the bottom view), the effective thin film area 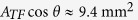
(projected area of satellite bubbles within the wedge shaped microlayer from Fig. 3a), the film thickness *H* when *F*_*D*_ = *F*_*UP*_ is predicted to be ≈28.6 nm. Since *H* of 28.6 nm is significantly larger than the critical film thickness *H*_*CR*_ for the rupture of the thin-film for DTAB (≈4 nm) and SDS (≈2 nm), the model predicts primary bubble departure, completely in agreement with the observations in our experiments (Supplementary Movie 4). For the range of bubble sizes observed in our experiments, the disjoining pressure at rupture (Fig. 3b), both for SDS and DTAB, is approximately three orders of magnitude higher than the buoyancy and surface tension combined. We believe that this high pressure is responsible for the large initial velocities of the departing bubbles in our experiments (Supplementary Movie 2). For the non-ionic surfactants, the repulsive interactions are relatively weaker^30^, and hence, the bubble departure frequency in our work is relatively smaller.

## Heat Transfer

**Figure 4:**
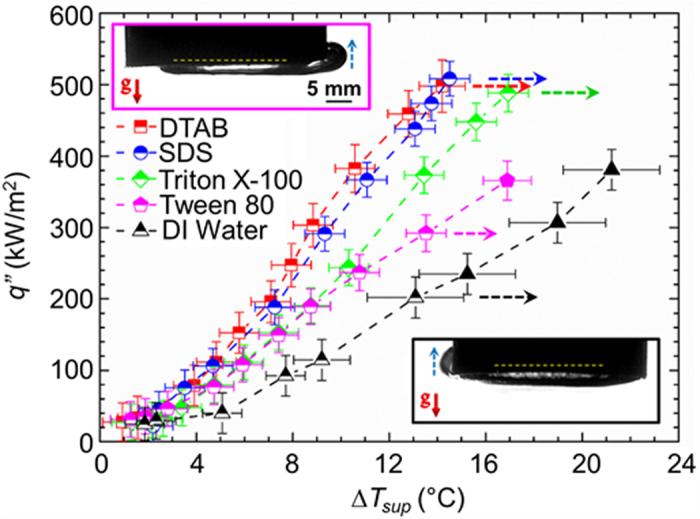
Pool boiling curves and sideways bubble departure. Plot of heat flux versus wall superheat with water and various aqueous surfactant solutions at CMC. Completely filled symbols correspond to heat transfer data with negative gravity induced sideways bubble departure. Images elucidating sideways departure (dotted blue arrow) of the big primary bubble for water (below) and Tween 80 (top) are shown in inset. Liquid pool was maintained at a temperature of 50 ± 1 °C.

Typical pool boiling curves (heat flux *q*″ versus superheat, *i.e.*, wall temperature rise above saturation, 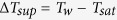) for DI water and various surfactants at critical micelle concentration (CMC) are shown in Fig. 4 (half-filled symbols). Horizontal dashed arrows indicate the maximum heat flux before the temperature continues to increase unabated (thermal runaway). Clearly, the maximum heat flux of ≈500 kW/m^2^ with DTAB, SDS, and Triton X-100 was significantly larger (≈2.4×) than that for DI water. Please note that few data points for DI water and Tween 80 in Fig. 4 extend beyond (filled symbols) the maximum heat flux values represented by the horizontal dashed arrows. In these cases, the primary bubble grew larger than the combined size of the heater surface and the surrounding Teflon insulation layer, resulting in sideways departure (images in inset of Fig. 4 and Supplementary Movie 1) due to negative buoyancy (*w.r.t.* downward facing/inverted heater configuration). The resulting enhancement in the maximum heat flux is an artefact of the current experimental scheme and will not be observed either if the experiments are performed in zero gravity, or a relatively large insulation layer is used to cover the heated block. Accordingly, the data points represented with solid symbols (beyond the maximum heat flux) are clearly an overestimation of the true potential of boiling in zero gravity where thermal runaway will be observed earlier (dashed arrow) for Tween 80 and water. Nonetheless, the limiting heat flux of ≈370–380 kW/m^2^ for water in our work is in close agreement with literature wherein the maximum value of ≈340 kW/m^2^ for the critical heat flux^31^ on inverted heaters was also attributed to the sideways bubble departure.

The corresponding HTC versus heat flux plots for the results presented in Fig. 4 are shown in Supplementary Fig. S3. The maximum HTC of ≈36 kW/m^2^-K in our work is significantly larger (1.9×) than the previously reported highest value of ≈19 kW/m^2^-K in literature^32^ (with water). Moreover, this value also corresponds to a significant (2.4×) enhancement in HTC in comparison to our experiments with pure water (15 kW/m^2^-K without sideways departure). Please note that the relative improvement of ≈2.4× in maximum heat flux and HTC in comparison to pure water is still an underestimation of the true potential of this approach for zero gravity due to the counteracting effect of buoyancy (increased thermal resistance due to multi-layered bubble structure and reduction in the bubble departure frequency due to the counteracting role of buoyancy) in our earth gravity experiments with inverted heaters.

Surfactants increase the nucleation site density, decrease the bubble size to increase the bubble removal frequency, and improve heat transfer coefficients during boiling on upward facing heaters in earth gravity as well^22^,^23^,^33^,^34^,^35^,^36^. However, the heat transfer coefficient enhancements in comparison to pure water are not as drastic as in this work since buoyancy on an upward facing heater by itself is strong enough to facilitate bubble departure. In addition, no enhancement in maximum heat flux is usually observed. In this regard, the role of surfactants as the sole bubble departure mechanism makes the enhancement in heat transfer coefficient and the maximum heat flux more pronounced in comparison to pure water on inverted heaters (or in zero gravity) where a single coalesced bubble as big as the heater is formed otherwise.

**Figure 5:**
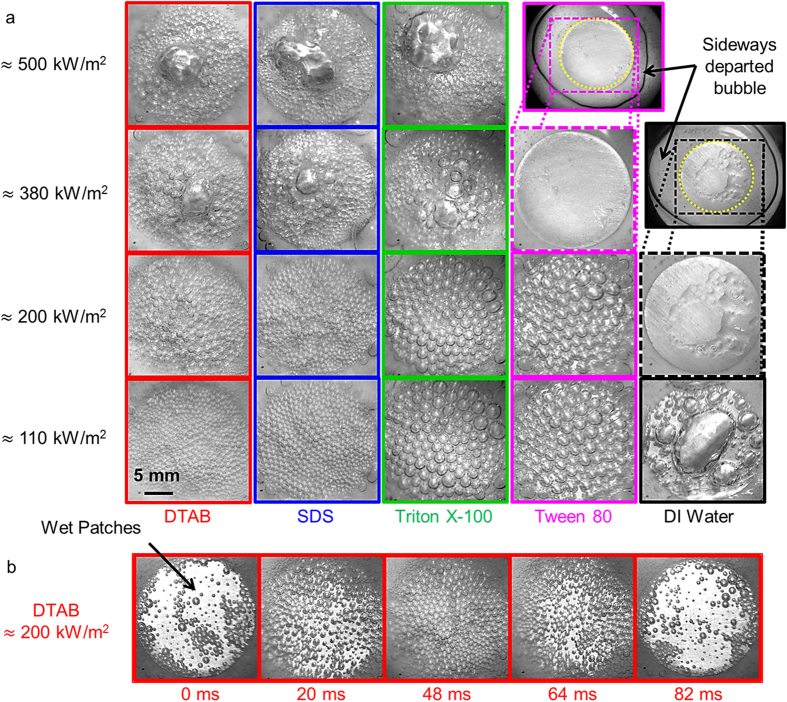
Single frame and time-lapse bottom view images. (**a**) Bottom view images of bubbles at different heat fluxes during subcooled pool boiling on an inverted heater. (**b**) Time-lapse image of bubble behaviour for one ebullition cycle with DTAB at a heat flux of ≈ 200 kW/m^2^. Gradual increase in empty wet patches between 48 ms to 82 ms signifies successive bubble removal. The bubble departure frequency was ≈12 Hz and the liquid pool temperature was maintained at 50 ± 1 °C.

To understand the fundamental mechanism of heat transfer enhancement, it is important to elucidate the effect of surfactant type on bubble behaviour and heat transfer. Bottom view images of bubbles for the heat transfer data presented in Fig. 4 are shown in Fig. 5a. These images were digitized and processed to estimate the corresponding values for the size distribution (average diameter *D*_*mean*_, minimum diameter *D*_*min*_, and the maximum diameter *D*_*max*_) and the number of bubbles (NOB) (Table 1). For pure water, a large non-departing primary bubble with a significant dry patch on the heater surface can be observed (images with black outline). The size of the bubble and the accompanying dry patches increased with heat flux. With the addition of surfactants, the bubble size was significantly reduced, contact line length increased, and wet patches were formed on the heater surface (Fig. 5a). At low heat fluxes, DTAB and SDS were most effective in minimizing coalescence and the resulting bubble size (Fig. 5a) were significantly smaller (<1 mm) than the heater diameter of 21.5 mm. Relatively large bubbles with diameters >1 mm were formed with TX-100 and Tween 80, nonetheless, much smaller than the heater size.

Coalescence was observed more frequently and the bubble size increased upon increasing the heat flux^37^. For example, the bubble behaviour at a heat flux of ≈380 kW/m^2^ for Tween 80 was similar to water where a big coalesced bubble was formed on the heater surface and sideways departure was reported. Assuming that the time *τ*_*D*_ required for surfactants to diffuse from bulk and adsorb at the liquid-vapor interface to avoid bubble coalescence was independent of heat flux, larger heat flux implied faster bubble growth rate and hence bigger bubbles (*D*_*max*_ in Table 1). Moreover, high heat flux also makes it relatively easier to evaporate the thin-liquid film between bubbles and force coalescence.

The bubble size also increased with heat flux for the case of DTAB, SDS, and Triton X-100, however, marginally in comparison to Tween-80. This behaviour can be related to the diffusion time scales *τ*_*D*_ of the surfactants^37^,^38^,^39^ (Supplementary Table S3). Diffusion time scales of milliseconds or smaller for DTAB, SDS, and Triton X-100 ensured faster adsorption of surfactants to the liquid-vapor interface. Accordingly, the tendency to coalesce was minimized and smaller bubbles were formed even at very high heat fluxes. However, significantly large diffusion time scale *τ*_*D*_ for Tween 80 was not sufficient to ensure enough concentration of surfactants at the interface required to avoid coalescence. As a result, large bubbles formed and the maximum heat flux (without sideways departure) was significantly lowered.

Bubble removal mechanism reported in Fig. 2b–d is further illustrated through the time-lapse images of the region of interest. One ebullition cycle of gradual bubble nucleation throughout the heater surface (0–48 ms) followed by surfactant-induced removal (48–82 ms) for DTAB is shown in Fig. 5b. This process of bubble removal allowed rewetting and the resulting increase in wet patches improved the heat transfer.

## Discussion

Heat transfer enhancement reported in boiling studies are often highly sensitive to contamination upon exposure to ambient. Little contamination is reported to gradually deteriorate wettability and lower the heat transfer. As a result, thorough cleaning procedures are often reported and the experiments are usually performed in highly controlled environment. While such efforts are required to understand the fundamental mechanism of boiling heat transfer, they are not practical for large-scale applications wherein it may not be practical to maintain pristine working environments. In this regard, we tested the robustness of our approach by performing two sets of experiments with DTAB and water (one in the morning and one in the evening) spanning over two hours each and continuing for a total of four consecutive days (*i.e.* a total of eight tests). Surface polishing and cleaning procedure similar to the experiments presented earlier were adopted before the start of the first experiment on day one. However, no further cleaning was performed for the next four days and experiments were continued in a relatively unclean environment of a mechanical workshop. The boiling chamber was open to ambient, accumulating significant contaminants, both on the heater surface and in test fluid towards the end of tests by day four. No significant change either in the bubble behaviour or any deterioration in the maximum heat flux (Supplementary Fig. S4) was observed with surfactant solutions over these eight tests spanning four days. The little decrease in HTC can be attributed to the increase in thermal resistance due to the deposition of a thick layer of contaminants including surfactants on to the heater surface. Closed boiling system with nominal cleaning practices will be enough to ensure that the high heat transfer coefficient values are also maintained for relatively longer durations.

All the experiments reported thus far were performed at CMC and the liquid pool temperature was maintained at 50 °C. We performed additional experiments where at CMC, the temperature of the liquid pool was changed to 70 °C and 90 °C (Supplementary Fig. S5). Conversely, another set of experiments were performed wherein the concentration of surfactants was lowered to half (CMC/2) and the liquid pool temperature was maintained at 50 °C (Supplementary Fig. S6). Surfactant-aided bubble removal on inverted heater was visually confirmed during each of these tests. Accordingly, boiling heat transfer was always improved in comparison to pure water (Supplementary Fig. S5 and S6).

In summary, we have presented a novel bubble removal mechanism that can help improve boiling heat transfer in the absence of buoyancy in space. In the future, it would be interesting to further investigate the effects of Marangoni convection due to concentration and temperature gradients^40^ at the liquid vapour-interface on the bubble departure mechanism. Furthermore, it would be interesting to evaluate the potential of this novel bubble departure mechanism for heat transfer enhancements during flow boiling conditions^41^. Although flow induced bubble removal during boiling with pure water is possible in zero gravity, the significantly reduced average bubble size in our experiments (Table 1) may drastically lower the contact line adhesion to the surface, increase the drag force (due to the reduced size), and result in easier bubble removal. Reduction in bubble size can delay dryout and earth gravity boiling heat transfer in confined spaces (mini-/micro- gaps or channels) may also be improved. We believe that the valuable insights presented here has implications for understanding bubble behaviour during other applications including foam and emulsion stabilization^42^, acoustic cavitation, sonochemistry and sonoluminescence^43^ as well.

## Methods

### Solution preparation

As received water soluble surfactants (Sigma Aldrich), namely, Dodecyltrimethylammonium Bromide (DTAB, CAS Number 1119-94-4), Sodium Dodecyl Sulphate (SDS, CAS Number 151-21-3), Polyoxyethylated t-octylphenol(Triton X-100, CAS Number 9002-93-1), and Polyethylene Sorbitan Monooleate (Tween 80, CAS Number 9005-65-6) were used in this work. The critical micelle concentration (CMC) of DTAB, SDS, Triton X-100 and Tween 80 are 4620 ppm, 2500 ppm, 200 ppm, and 15 ppm, respectively. The glassware was cleaned with acetone and thoroughly washed with water before preparing the surfactant solution. Surfactants were added in DI water and the solution was stirred for 1 hour using magnetic stirrer. The aqueous surfactant solution was then stored for one day before starting the boiling experiment. Experiments were performed at CMC and 0.5CMC for each surfactant. The relevant physico-chemical properties of the four surfactants used are shown in Supplementary Table S1.

### Heater surface preparation

Prior to the start of each round of experiments, the aluminum heater surface was polished with a 600 grit size sand paper. The complete heater assembly was next cleaned with acetone followed by thorough DI water wash.

### Experiments

A sectional view of the computer aided design (CAD) model of the experimental setup is shown in Supplementary Fig. S1. Subcooled pool boiling experiments (liquid pool temperature 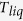 was lower than the saturation temperature 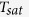
of water at ambient pressure, 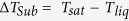) were performed at three subcoolings of 50 ± 1 ^o^C (high subcooling), 30 ± 1 ^o^C (intermediate subcooling), and 10 ± 1 ^o^C (low subcooling). Heating of the Aluminum block was initiated using the DC power source and the wall temperature corresponding to each supplied voltage was monitored for steady-state conditions. After each data run, the voltage was increased in steps and the steady-state data for the next level of the input power was acquired.

At low heat fluxes, there was negligible temperature gradient in the bulk liquid, *i.e.*, insignificant difference in the temperatures recorded by thermocouples *T*_*1*_ and *T*_*2*_. While a significant increase in the temperature of thermocouple *T*_*2*_ was observed at high heat flux values, the temperature of thermocouple *T*_*1*_ was changed insignificantly. As a result, the 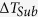 in our experiments were based on the 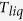 measured by thermocouple *T*_*1*_.

### Data Reduction

Time-averaged **s**teady–state temperature of thermocouples *T*_*4*_, *T*_*5*_, and *T*_*6*_ were used with Fourier’s law of conduction (one-dimension) to estimate the experimental heat flux. The time-averaged temperature *T*_*3*_ measured at a height of 2.5 mm above the inverted heater surface was extrapolated to find the actual temperature at the boiling surface (*T*_*w*_). Three dimensional steady-state heat conduction simulations of the heater assembly were performed using COMSOL^TM^ software (aluminium block and Teflon insulation) for estimating the heat loss to the ambient and insulation. Input power was assigned to the cartridge heater of the COMSOL^TM^ model as uniform volumetric heat generation. Heat transfer coefficient of 1000 W/m^2^-K and 10 W/m^2^-K^44^ were assigned as the boundary condition to the heater assembly for natural convection with water and air, respectively. Heat transfer coefficient of the boiling surface was varied until the wall temperature *T*_*w*_ matched the extrapolated temperature at the heated surface. The predicted value of heat flux at this value of temperature for the boiling surface was found to be in good agreement (Supplementary Fig. S2) with the heat flux values estimated using the temperature gradients measured with thermocouples *T*_*4*_, *T*_*5*_, and *T*_*6*_. The bubble removal/departure frequency was estimated by taking the average of 20 removal events each recorded from the side view and the bottom view images.

### Uncertainty Analysis

An uncertainty of 0.5 °C in the thermocouple reading and the standard deviation of the time-averaged temperature data over the steady-state period were used to estimate the root mean square error (RMSE) in the temperatures. Similarly, the uncertainty in heat flux was estimated using the uncertainties associated with the thermocouples used for heat flux estimation. The uncertainty in heat transfer coefficient was dependent on the uncertainty in heat flux and wall superheat. The surfactant concentration varied during experiments due to the evaporation of water. Based on the variation in the liquid column level, the uncertainty in the concentration (for experiments at CMC) was estimated to be 60 ppm, 32 ppm, 3 ppm and 1 ppm for DTAB, SDS, Triton X-100, and Tween 80, respectively. The uncertainty in the bubble removal/departure frequency was based on the statistical deviation between 20 measurements at each test condition.

## Additional Information

**How to cite this article**: Raza, MQ. *et al.* Surfactants for Bubble Removal against Buoyancy. *Sci. Rep.* **6**, 19113; doi: 10.1038/srep19113 (2016).

## Supplementary Material

Supplementary Movie 1

Supplementary Movie 2

Supplementary Movie 3

Supplementary Movie 4

Supplementary Information

## Figures and Tables

**Table 1:** Number and size distribution (mm) of bubbles with heat flux.

*q*″ 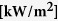	DTAB	SDS	Triton X-100	Tween 80	
≈500	1.18 (0.58–8.65)	0.94 (0.51–10.2)	1.15 (0.85–8.84)	NA	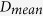 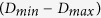
	200 ± 20	220 ± 20	190 ± 20	1	NOB
≈380	1.25 (0.57–5.67)	1.15 (0.5–4.15)	1.54 (0.86–6.45)	NA	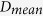 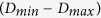
	250 ± 30	250 ± 20	125 ± 20	1	NOB
≈200	0.83 (0.51–1.44)	0.81 (0.50–1.17)	1.48 (0.7–2.5)	1.75 (1.06–3.00)	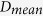 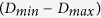
	400 ± 20	410 ± 20	130 ± 10	100 ± 10	NOB
≈110	0.75 (0.51–1.09)	0.82 (0.56–1.06)	1.41 (0.58–2.75)	1.67 (0.99–2.22)	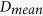 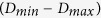
	550 ± 20	425 ± 20	140 ± 10	110 ± 10	NOB

Liquid pool temperature was maintained at 50 ± 1 °C.
