# Supplementary material for: Surfactants for Bubble Removal against Buoyancy
Source: Sci Rep. 2016 Jan 8;6:19113. doi: 10.1038/srep19113 (PMC4705484; doi:10.1038/srep19113)
Supplement: Supplementary Information [file srep19113-s5.pdf]

# Supplementary Information

## Surfactants for Bubble Removal against Buoyancy

*Md. Qaisar Raza, Nirbhay Kumar, and Rishi Raj*[1]

*Thermal and Fluid Transport Laboratory, Department of Mechanical Engineering,*

*Indian Institute of Technology Patna, Bihar 800013, India*

## Supplementary Figures

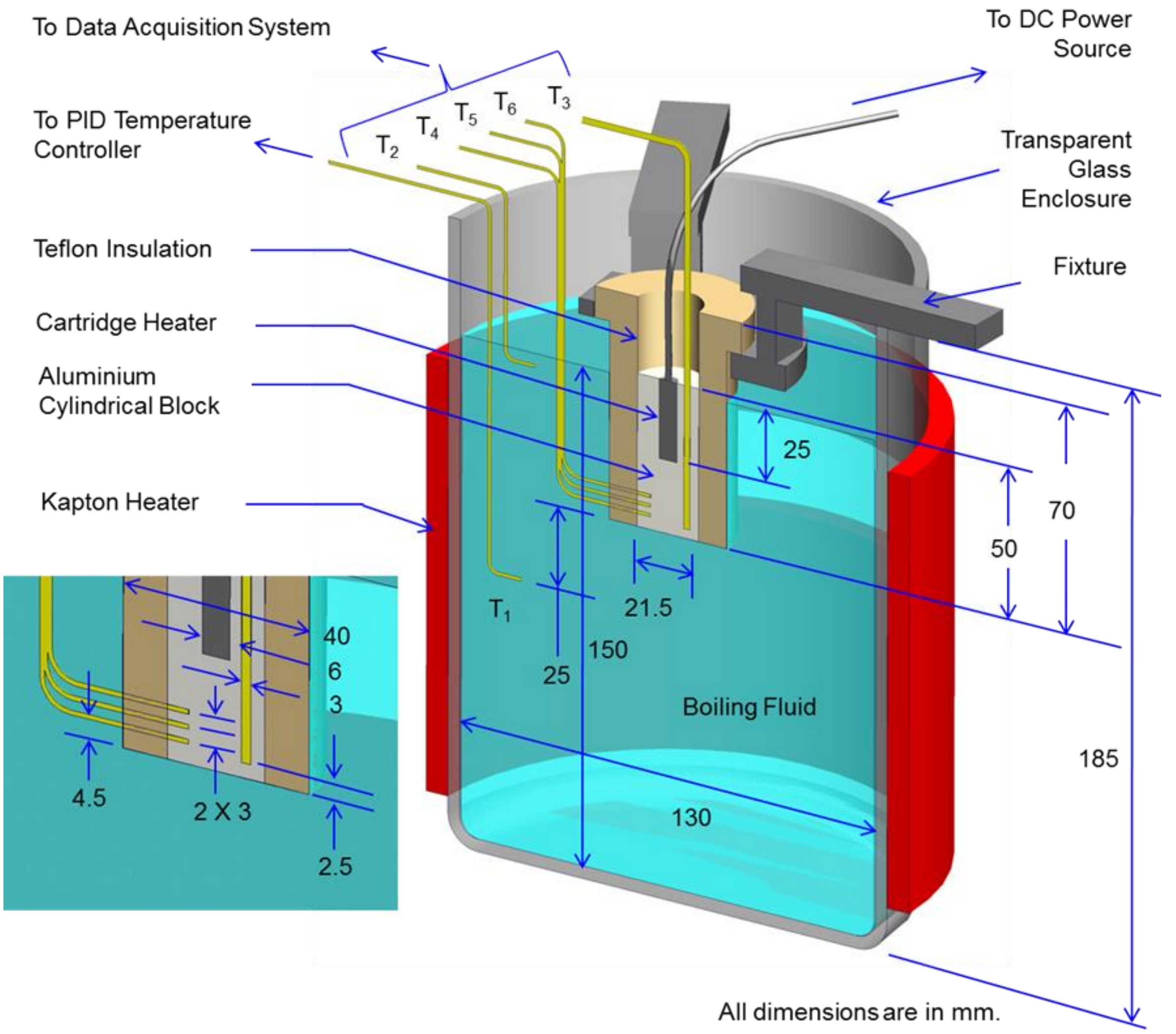


**Supplementary Figure S1 | Experimental Setup.** A transparent cylindrical enclosure contains the test fluid along with an inverted aluminum heater block assembly. The aluminum block was insulated by a Teflon bush of 40 mm outer diameter. The cartridge heater (250W,

[1] *Author for correspondence:* rraj@iitp.ac.in

110V, Make-Marathon) was embedded inside the aluminum block. A DC power source (Agilent, N5751A) was used to supply power to the cartridge heater. Thermal grease was used to minimize the contact resistance and provide good heat transfer to aluminum block. Input power and temperatures of various thermocouples were then acquired and the bubble behavior was captured from the side and bottom using the high speed camera. Thermocouples $T_3$, $T_4$, $T_5$, and $T_6$ (J-type) were used for estimating the heater surface temperature and heat flux. The liquid column was kept open to ambient and temperature of the pool was controlled using the kapton heater wrapped around the cylindrical enclosure, the thermocouple $T_1$, and a PID controller.

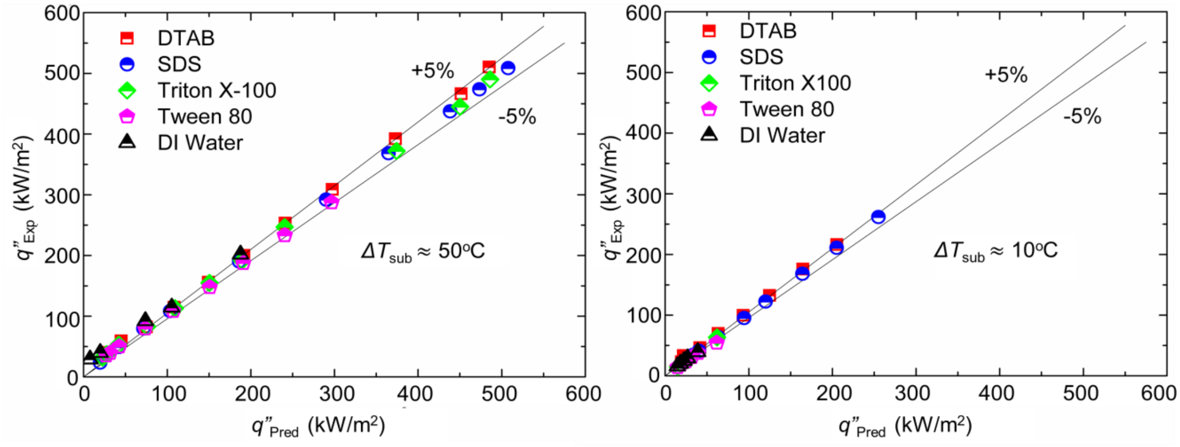


**Supplementary Figure S2 | Experimental versus predicted heat flux.** Comparison of the experimentally measured and predicted heat flux values at two subcoolings. Good agreement suggests that the heat loss to the insulation and the fixture have been estimated accurately in our experiments.

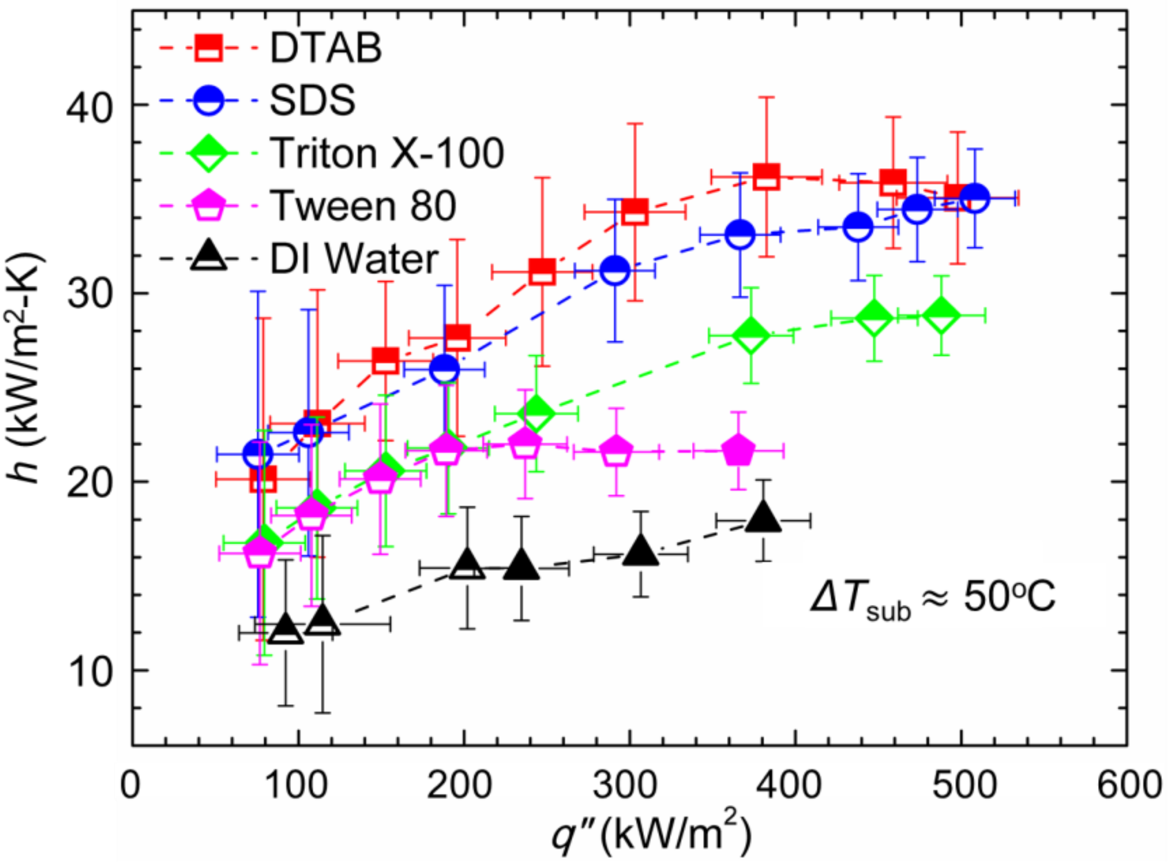


**Supplementary Figure S3 |Heat transfer coefficient versus heat flux.** The data is this plot corresponds to the heat flux versus superheat plot in Fig. 4 of the main text. The maximum HTC of ≈36 kW/$m^2$-K with DTAB is significantly larger (2.4×) than the highest value of ≈15 kW/$m^2$-K (without sideways departure) for pure water.

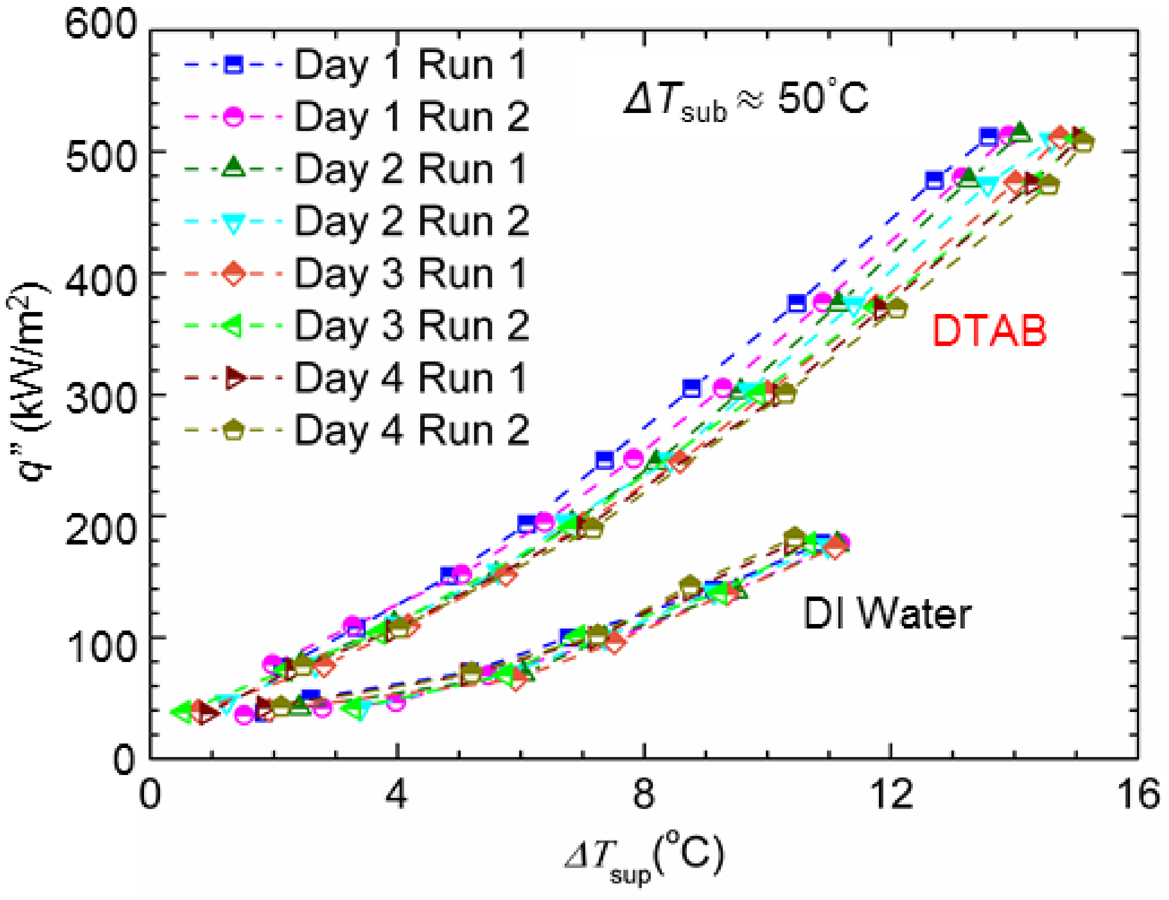


**Supplementary Figure S4 |Robustness.** Subcooled pool boiling curves for water and DTAB solutions. Two sets of experiments with DTAB and water (Run 1 in the morning and Run 2 in the evening) spanned over two hours each and continued for a total of four consecutive days (Day 1-4). Negligible deterioration in the maximum heat flux was observed over these eight tests spanning four days. The little decrease in the heat transfer coefficient for DTAB can be attributed to the relative increase in thermal resistance due to the deposition of contaminants including surfactants at the boiling surface.

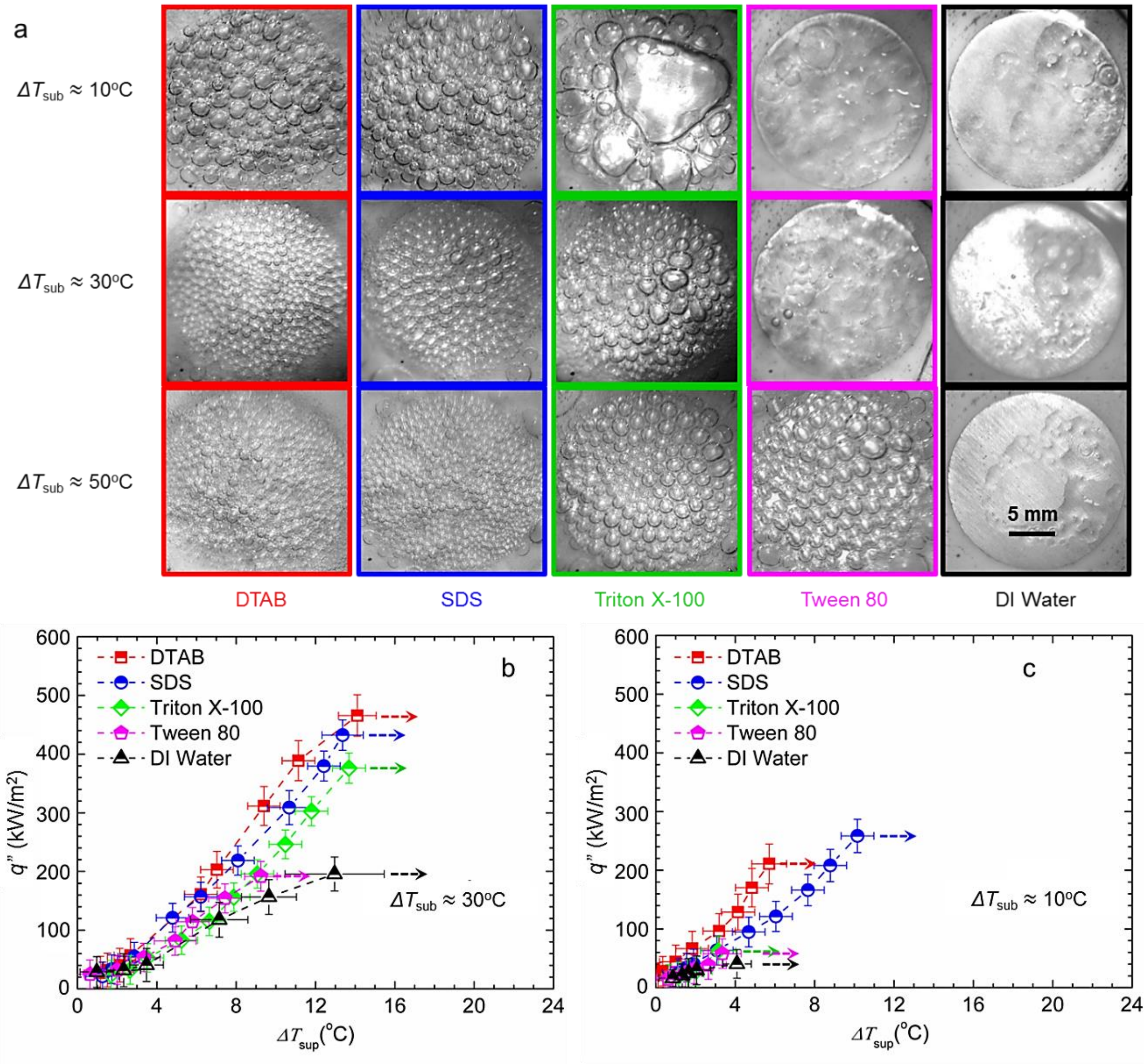


**Supplementary Figure S5 |Effect of subcooling. (a)** Bottom view images of bubbles at different subcoolings and at a heat flux of ≈200 kW/m$^2$. Size of the bubble increased with decreasing subcooling. At all subcoolings tested, a big primary bubble which covered the entire heater area was formed during boiling with pure water. In case of Tween 80, a big primary bubble was formed at low and intermediate subcoolings of 30±1°C and 10±1°C. In case of SDS and DTAB, size of the bubbles increased marginally upon lowering the subcooling. **(b)** Subcooled pool boiling curves at intermediate subcooling. **(c)** Subcooled pool boiling curves at low subcooling. Data corresponding to sideways departure (similar to solid symbols in Fig. 3, main text) for water and Tween 80 has not been shown.

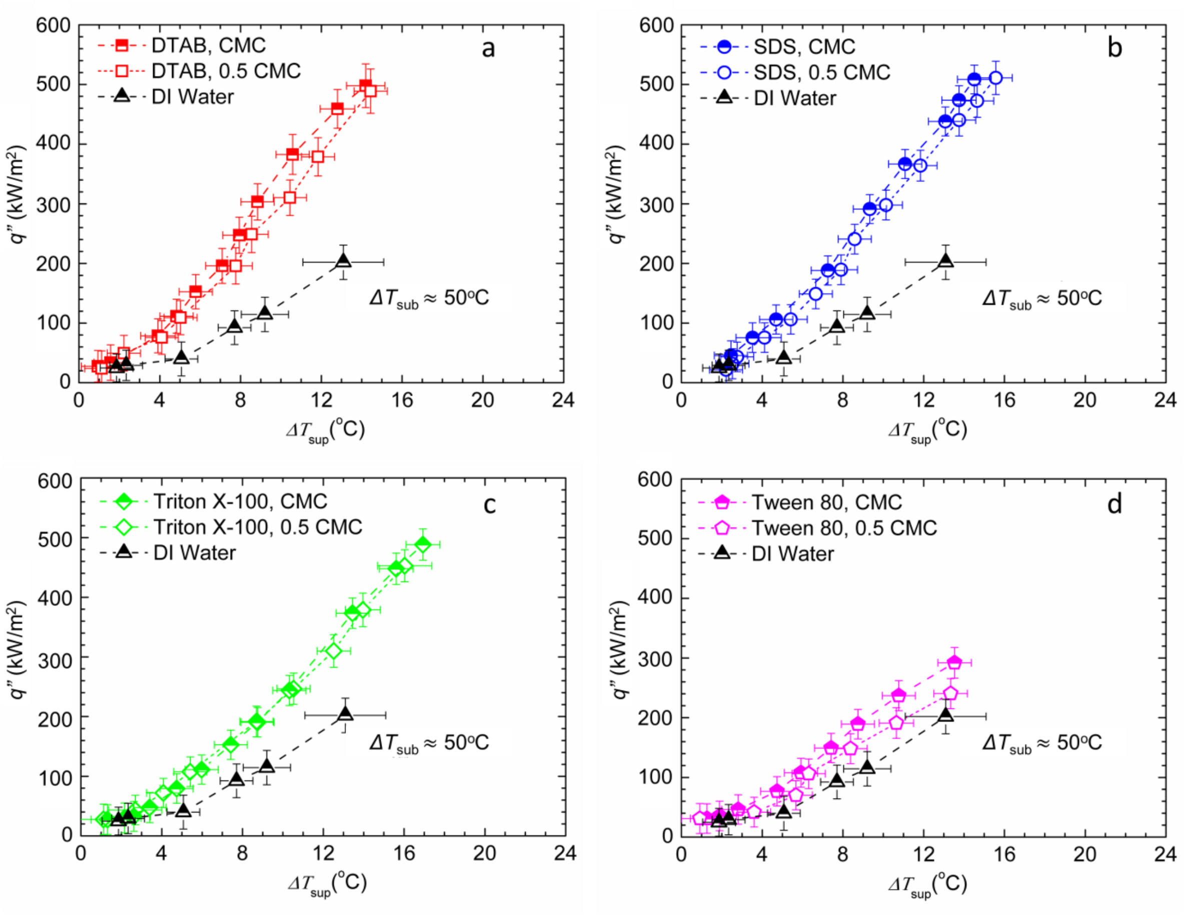


**Supplementary Figure S6 | Effect of surfactant concentration.** Plot of heat flux versus wall superheat with water and various aqueous surfactant solutions at two concentrations (0.5CMC and CMC). At both concentrations, significant enhancement in both, the heat transfer coefficient and the maximum heat flux was observed in comparison to water. Aqueous surfactant solutions at CMC always performed better in comparison to lower concentration of 0.5CMC. Liquid pool was maintained at a temperature of 50±1°C.

# Supplementary Tables

**Supplementary Table S1:** Physico-chemical properties of surfactant.

| Surfactants | DTAB | SDS | Triton-X100 | Tween 80 |
|---|---|---|---|---|
| Chemical formulae | $CH_3(CH_2)_{11}N(CH_3)_3Br$ | $C_{12}H_{25}SO_4Na$ | $C_4H_{21}(OCH_2CH_2)_{9-10}OH$ | $C_{64}H_{124}O_{26}$ |
| Ionic Nature | Cationic | Anionic | Non-ionic | Non-ionic |
| Form | White powder | White powder | Clear Liquid | Liquid |
| Molecular weight | 308.34 | 288.3 | 624 | 1310 |
| Specific gravity | - | 0.4 | 1.065 | 1.07 |
| CMC | ~ 4620 ppm | ~ 2500 ppm | ~ 200 ppm | ~ 15 ppm |

**Supplementary Table S2:** Value of various parameters used for the estimation of disjoining pressure.

| **Parameters** | **SDS**[26] | **DTAB**[27] |
|---|---|---|
| $T$ (K) | 373.15 | 373.15 |
| $C_S$ (mol/lit) | $8.6715 \times 10^{-3}$ | $14.98 \times 10^{-3}$ |
| $\theta$ (°) | 30 | 30 |
| $\sigma$ (N/m) | 0.037 | 0.040 |
| $\psi_s$ at CMC (mV) | $-123$ | 50 |
| $z$ | 1 | 1 |
| $A_{232}$ (J) | $3.7 \times 10^{-20}$ | $3.7 \times 10^{-20}$ |
| $K_{232}$ at CMC (J) | $-2.09 \times 10^{-20}$ | ……… |

**Supplementary Table S3**: Characteristic time scales of diffusion ($\boldsymbol{\tau_D = \Gamma_{eq}^2/C^2D}$) reported/calculated from the data available in literature.

| Surfactants | $\boldsymbol{\Gamma_{eq}}$ (mol/m$^2$) | $\boldsymbol{C}$ (mol/m$^3$) | $\boldsymbol{D}$ (m$^2$/s) | $\boldsymbol{\tau_D}$ | References |
|---|---|---|---|---|---|
| DTAB | 9.00 X $10^{-7}$ | 14.9186 | 4.0 X $10^{-10}$ | ~ 0.14 ms | Carey & Stubenrauch (2009)[37] |
| SDS | 6.27 X $10^{-6}$ | 8.6715 | 4.6 X $10^{-10}$ | ~ 1.13 ms | Wu *et al.* (1999) [38] |
| Triton X100 | 3.09 X $10^{-6}$ | 0.3205 | 3.7 X $10^{-10}$ | ~ 251 ms | Wu *et al.* (1999)[38] |
| Tween 80 | 2.76 X $10^{-6}$ | 0.0114 | 1.0 X $10^{-10}$ | ~ 580 s | Samanta *et al.* (2011)[39] |

# Supplementary Movies

**Supplementary Movie 1.** Boiling of pure water on an inverted heater. The temperature of the liquid pool was 50℃ and the heat flux was maintained at 380 kW/m$^2$.

**Supplementary Movie 2.** Boiling of aqueous surfactant solution (DTAB at CMC) on an inverted heater. The temperature of the liquid pool was 50℃ and the heat flux was maintained at 200 kW/m$^2$.

**Supplementary Movie 3.** Illustration of the bubble departure mechanism during boiling with aqueous surfactant solution (DTAB at CMC) on an inverted heater. Small satellite bubbles formed after the new nucleation events within the wedge shaped microlayer region of the relatively large primary bubble act as a precursor to departure. The temperature of the liquid pool was 50℃ and the heat flux was maintained at 200 kW/m$^2$.

**Supplementary Movie 4.** Illustration of the bubble departure mechanism during boiling with aqueous surfactant solution (DTAB at CMC) on an inverted heater. Small satellite bubbles formed after the new nucleation events within the wedge shaped microlayer region of the relatively large primary bubble act as a precursor to departure. The temperature of the liquid pool was 90℃ and the heat flux was maintained at 200 kW/m$^2$.
